# Evaluation of the Effects of *Cuminum cyminum* on Cellular Viability, Osteogenic Differentiation and Mineralization of Human Bone Marrow-Derived Stem Cells

**DOI:** 10.3390/medicina57010038

**Published:** 2021-01-04

**Authors:** Hyunjin Lee, Youngmin Song, Yoon-Hee Park, Md. Salah Uddin, Jun-Beom Park

**Affiliations:** 1 Department of Periodontics, College of Medicine, The Catholic University of Korea, Seoul 06591, Korea; hyunjinlee0423@gmail.com (H.L.); hahahhh99@naver.com (Y.S.); 2 Ebiogen, Seoul 07282, Korea; yhpark@e-biogen.com; 3 Ethnobotanical Database of Bangladesh, Tejgaon, Dhaka 1208, Bangladesh; plantsofbd@gmail.com

**Keywords:** *Cuminum*, herbal medicine, cell differentiation, cell survival, stem cells

## Abstract

*Background and Objectives: Cuminum cyminum* L. has long been used in the treatment of various diseases in multiple geographical regions. This study was performed to determine the effects of *C. cyminum* methanolic extract (CCT) on the cellular viability, alkaline phosphatase activity and mineralization of human mesenchymal stem cells. *Materials and Methods:* Bone marrow-derived stem cells were cultured in the presence of CCT at concentrations of 0, 0.001, 0.01, 0.1 and 1 μg/mL. Evaluations of cell morphology were performed on days 1, 3, 7 and 14. Cellular viability was evaluated on days 1, 3, 5 and 7. On the 7th and 14th day, alkaline phosphatase activity measurements and Alizarin red S staining were conducted to assess the osteogenic differentiation of stem cells. A real-time polymerase chain reaction was used to determine the expression levels of RUNX2, BSP, OCN, COL2A1 and β-catenin mRNAs. *Results:* Stem cells in the control group showed fibroblast-like morphology and the addition of CCT at 0.001, 0.01, 0.1 and 1 μg/mL did not generate noticeable changes in morphology compared with the untreated control group. The application of CCT did not produce significant changes in cellular viability or alkaline phosphatase activity compared with controls. Alizarin Red S staining was significantly increased with the application of CCT. Treatment with CCT increased the expressions of RUNX2, BSP and OCN. *Conclusions:* These results indicate that CCT enhanced the osteogenic differentiation of stem cells derived from bone marrow by regulating the expressions of RUNX2, BSP and OCN. Thus, the use of CCT may be applied to achieve beneficial effects on the mineralization of stem cells.

## 1. Introduction

*Cuminum cyminum* L. (cumin) has been used to treat various indications in various geographical regions [1]. Cumin is a rich source of essential oils and has been actively researched for its chemical composition and biological activities [1]. Cumin has been applied for the treatment of various diseases [2]. Cumin was shown to have an effect on insulin metabolism and it was effective on weight loss in overweight participants [3]. Cumin had some effects on participants with metabolic syndrome [4]. Moreover, cumin has been reported to lower the plasma lipid concentration in non-hypertriglyceridemia participants [5]. Additionally, cumin has been reported to have antioxidant, antiallergic and antiplatelet effects [6]. Cumin has been applied to treat cancer [7]. Cumin has been suggested to be used as antibacterial agent and it was also effective against *Candida* infections [8,9].

Stem cells are of great interest, especially to cure various diseases [10]. Stem cells have various functions [11]. Not only do stem cells have the ability to differentiate into various tissues, but they also secrete various factors [12]. Through this feature, called the paracrine effect, stem cells can affect the surrounding tissues [13]. Stem cells are currently being used in tissue regeneration [14]. The use of stem cells in bony defects has been shown to improve bone regeneration in mandibular defects [15]. Herbal extracts have been applied for the enhancement of functionality of stem cells [16,17]. In a previous study, *Bambusa tulda* extract increased the cell proliferation and collagen I expression of stem cells at early time points [17]. *Cimicifugae rhizome* extracts have been shown to increase osteogenic differentiation of gingiva-derived mesenchymal stem cells [18]. A paste was made with the seeds of the cumin plant and was applied for the treatment of cutting wounds. It was shown that the alcohol extract of the seeds of cumin promoted wound healing on excision, incision and granuloma wound models [19,20]. To the best of our knowledge, there are no previous studies evaluating the effects of cumin on bone marrow-derived stem cells. In light of the promising findings in previous studies on cumin, the aim of the present study was to examine the effects of cumin methanolic (CCT) extracts to maintain the cellular viability and enhance the mineralization of human mesenchymal stem cells.

## 2. Materials and Methods

### 2.1. Preparation of Plant Materials

*C. cyminum* L. was collected by Md. Salah Uddin from the Shibgonj sub-district, Bogra district, Rajshahi division in Bangladesh. Voucher samples were deposited in the herbarium of the Korea Research Institute of Bioscience and Biotechnology as KRIB 0086021. After drying and grinding the seeds of *C. cyminum,* the powder (75 g) was extracted by applying 1 L of 99.9% (*v*/*v*) methanol for 3 days at 45 °C. Sonication was performed for 15 min and resting was done for 2 h. The resultants were filtered with non-fluorescent cotton and concentrated at 45 °C by a rotary evaporator (N-1000SWD, EYELA, Tokyo, Japan) using reduced pressure. A total 13.16 g of CCT extract was obtained after freeze-drying procedures.

### 2.2. Study Design Using Bone Marrow Mesenchymal Stem Cells (BMSCs)

**Figure 1:**
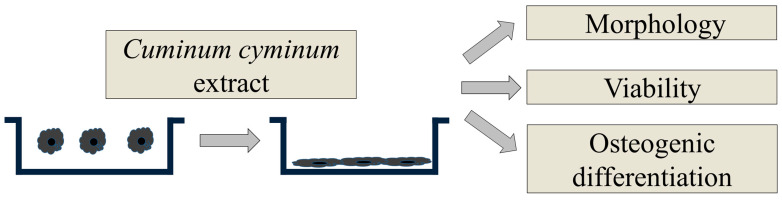
Schematic overview of the study design.

This research protocol was reviewed and approved by the Institutional Review Board of Seoul St Mary’s Hospital, College of Medicine, The Catholic University of Korea (KC19SISI0816 and KC20SISE0582, 20 November 2019). Human BMSCs (Catholic MASTER cells) were obtained from the Catholic Institute of Cell Therapy (CIC, Seoul, Korea). The cells were derived from human bone marrow donated by healthy donors after informed consent was procured from the male participants in their twenties. All experiments were performed based on the relevant guidelines and regulations specified in the Declaration of Helsinki. Figure 1 shows a general view of the research design. The isolation and characterization of BMSCs were carried out in accordance with a previous method [21]. The cells were plated on a culture plate and the cells that were not attached to the plate were eliminated. We changed the culture medium every two or three days. The cells were grown in an incubator at 37 °C with 95% air and 5% CO_2_. Three experimental repeats were evaluated for the analysis.

### 2.3. Evaluation of Cell Morphology

Stem cells were cultivated in in an osteogenic medium (alpha-minimal essential medium, α-MEM, Gibco, Grand Island, NY, USA) and 15% fetal bovine serum (Gibco) supplemented with 2 mg/mL of glycerophosphate disodium salt hydrate, 38 μg/mL of dexamethasone, 10 mM of ascorbic acid 2-phosphate, 200 mM of L-Glutamine (Sigma-Aldrich Co., St. Louis, MO, USA) along with penicillin and streptomycin (Sigma-Aldrich Co.). Cells were treated with CCT at final concentrations of 0, 0.001, 0.01, 0.1 and 1 μg/mL. The morphological evaluation was carried out on the 1st, 3rd, 7th and 14th day using an inverted microscope (CKX41SF, Olympus Corporation, Tokyo, Japan).

### 2.4. Evaluation of Cellular Viability

On days 1, 3, 5 and 7, the evaluation of cellular viability was performed using the Counting Kit-8 assay (CCK-8, Dojindo, Tokyo, Japan) following the manufacturer’s instructions [22]. In short, cells were incubated with tetrazolium monosodium salt for 1 h at 37 °C. Absorbance at 450 nm was detected spectrophotometrically using a microplate reader (BioTek Instruments Inc., Winooski, VT, USA).

### 2.5. Quantitative Assay of Alkaline Phosphatase Activity and Quantitative Detection of Alizarin Red S Staining

To access the osteogenic differentiation of stem cells, absorbance at 405 nm was measured using an alkaline phosphatase assay kit (K412-500, BioVision, Inc., Milpitas, CA, USA) according to the manufacturer’s protocol after 1, 3, 7 and 14 days of cell culture using a microplate reader (BioTek Instruments Inc.).

The cells were washed, fixed and colored with 2% Alizarin Red S Solution (ScienCell Research Laboratories, Inc., Carlsbad, CA, USA) after 7 and 14 days of cell culture. The stained cells were visualized using a microscope (CKX41SF, Olympus Corporation). Ten percent cetylpyridinium chloride (Sigma-Aldrich Co.) was used to dissolve the bound dye and quantification was performed spectrophotometrically at 560 nm.

### 2.6. Total RNA Extraction and Quantification of RUNX2, BSP, OCN and COL2A1 mRNA by Real-Time Polymerase Chain Reaction (PCR)

Total RNA extraction was performed using a commercially available kit (Thermo Fisher Scientific, Inc., Waltham, MA, USA) according to the manufacturer’s instructions [23]. The quality of RNA was evaluated with a bioanalyzer (Agilent 2100) using a kit (RNA 6000 Nano Chip; Agilent Technologies) and RNA quantity was evaluated with the ratio of absorbance at 260 nm and 280 nm using a spectrophotometer (ND-2000, Thermo Fisher Scientific, Inc.). RNA was used as a reverse transcription template applying reverse transcriptase (SuperScript II; Invitrogen, Carlsbad, CA, USA).

mRNA expression was detected by real-time PCR. We used GenBank to design the sense and antisense primers for the PCR. The primer sequences were as follows: RUNX2 (accession No.: NM_001015051.3; forward: 5′-CAGTTCCCAAGCATTTCATCC-3′, reverse: 5′-AGGTGGCTGGATAGTGCATT-3′), BSP (accession No.: NM_004967.4; forward: 5′-CCTCTCCAAATGGTGGGTTT-3′, reverse: 5′-ATTCAACGGTGGTGGTTTTC-3′), OCN (accession No.: NM_199173.6; forward 5′-GGTGCAGAGTCCAGCAAAGG-3′, reverse: 5′-GCGCCTGGGTCTCTTCACTA-3′), COL2A1 (accession No.: NM_033150.3; forward: 5′-AAGGTTTTCTGCAACATGGA-3′, reverse: 5′-TCTTCTTGGGAACGTTTGCT -3′) and β-actin (accession. No.: NM 001101: forward: 5′-AATGCTTCTAGGCGGACTATGA-3′, reverse: 5′-TTTCTGCGCAAGTTAGGTTTT-3′). Normalization was performed using the β-actin housekeeping gene. Real-time PCR was performed using the SYBR Green PCR Kit (Applied Biosystems, Waltham, MA, USA) on the PCR System (StepOnePlus^TM^; Applied Biosystems) following the manufacturer’s recommendations.

### 2.7. Statistical Analysis

Data are displayed as means ± standard deviations of the experiments. A normality test was performed and one-way analysis of variance with a post hoc Tukey’s test was used to compare the results between the groups using a computer-based statistical and computational software (SPSS 12 for Windows, SPSS Inc., Chicago, IL, USA). The level of significance was set at 0.05.

## 3. Results

### 3.1. Evaluation of Cell Morphology

**Figure 2:**
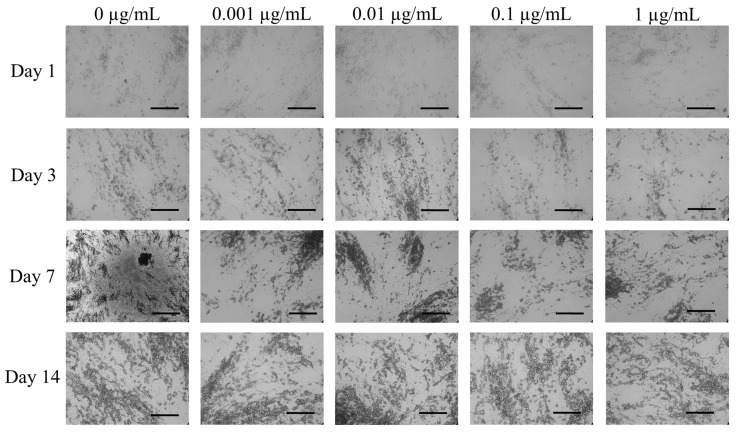
Evaluation of cell morphology on days 1, 3, 7 and 14 using inverted microscopy following treatment with different concentrations of cumin methanolic (CCT) in osteogenic media (original magnification ×100). Scale bars indicate 200 μm.

Figure 2 shows the morphology of BMSCs treated with CCT at the final concentrations of 0, 0.001, 0.01, 0.1 and 1 μg/mL on the first day. The stem cells in the 0 ng/mL group on the first day showed fibroblast-like morphology. The morphology of the stem cells of the 0.001, 0.01, 0.1 and 1 μg/mL groups did not show significant changes compared with the untreated control group. Extended incubation to days 3, 7 and 14 did not result in any morphological changes.

### 3.2. Cellular Viability

**Figure 3:**
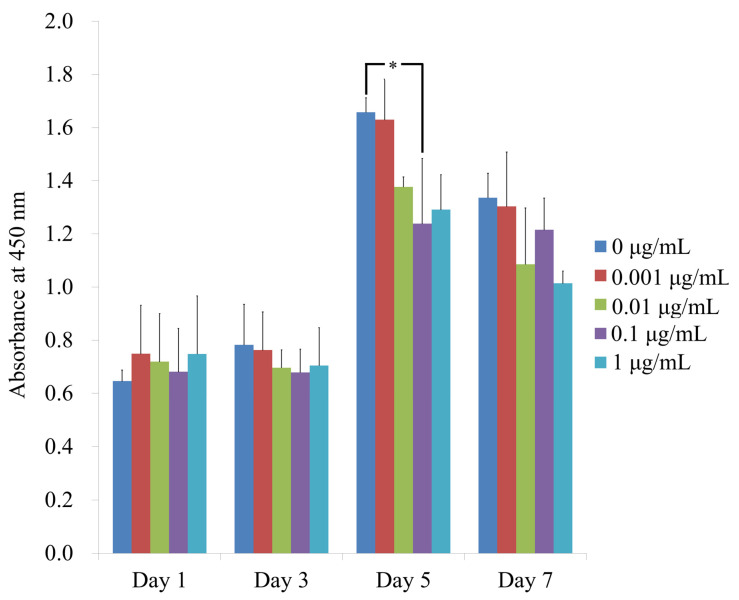
Evaluation of cellular viability using CCK-8 assay on days 1, 3, 5 and 7. * Statistically significant differences compared with CCT at 0 μg/mL on day 5 (*p* < 0.05).

The CCK-8 assay results for cellular viability performed on days 1, 3, 5 and 7 are shown in Figure 3. The absorbance values at 450 nm on day 1 in cells treated with 0, 0.001, 0.01, 0.1 and 1 μg/mL CCT were 0.646 ± 0.041, 0.749 ± 0.182, 0.719 ± 0.181, 0.681 ± 0.163 and 0.748 ± 0.218, respectively. No statistically significant differences in cell viability were observed between the groups (*p* > 0.05). The absorbance values on day 5 in cells treated with 0, 0, 0.001, 0.01, 0.1 and 1 μg/mL CCT were 1.658 ± 0.054, 1.629 ± 0.153, 1.377 ± 0.037, 1.238 ± 0.245 and 1.291 ± 0.132, respectively. CCT at 0.1 μg/mL showed statistically significant differences when compared with CCT at 0 μg/mL on day 5 (*p* < 0.05). The absorbance values on day 7 in cells treated with 0, 0, 0.001, 0.01, 0.1 and 1 μg/mL CCT were 1.336 ± 0.092, 1.303 ± 0.204, 1.086 ± 0.212, 1.215 ± 0.120 and 1.014 ± 0.046, respectively (*p* > 0.05).

### 3.3. Alkaline Phosphatase Activity Assays

**Figure 4:**
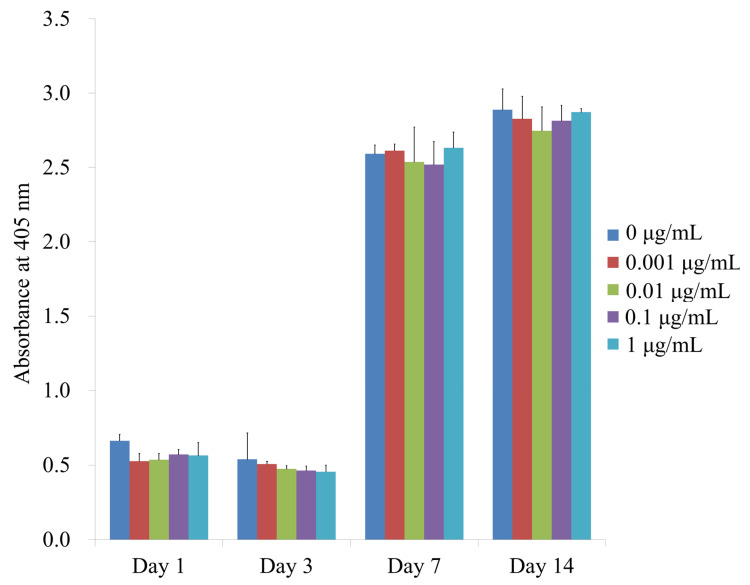
Alkaline phosphatase activity on days 1, 3, 7 and 14.

The alkaline phosphatase activity of cells treated with CCT at days 1, 3, 7 and 14 is shown in Figure 4. The absorbance values at 405 nm in cells cultured with 0, 0.001, 0.01, 0.1 and 1 μg/mL CCT on day 7 were 2.591 ± 0.059, 2.613 ± 0.044, 2.537 ± 0.234, 2.519 ± 0.154 and 2.632 ± 0.105, respectively (*p* > 0.05). The absorbance values in cells cultured with 0, 0.001, 0.01, 0.1 and 1 μg/mL CCT on day 14 were 2.888 ± 0.141, 2.827 ± 0.151, 2.746 ± 0.160, 2.814 ± 0.105 and 2.872 ± 0.024, respectively (*p* > 0.05). There were no significant differences between the groups in each time point.

### 3.4. Mineralization Assay

**Figure 5:**
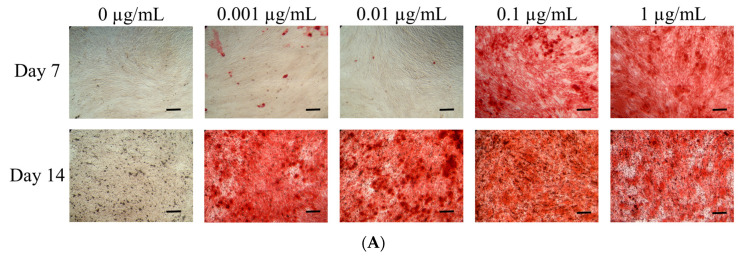

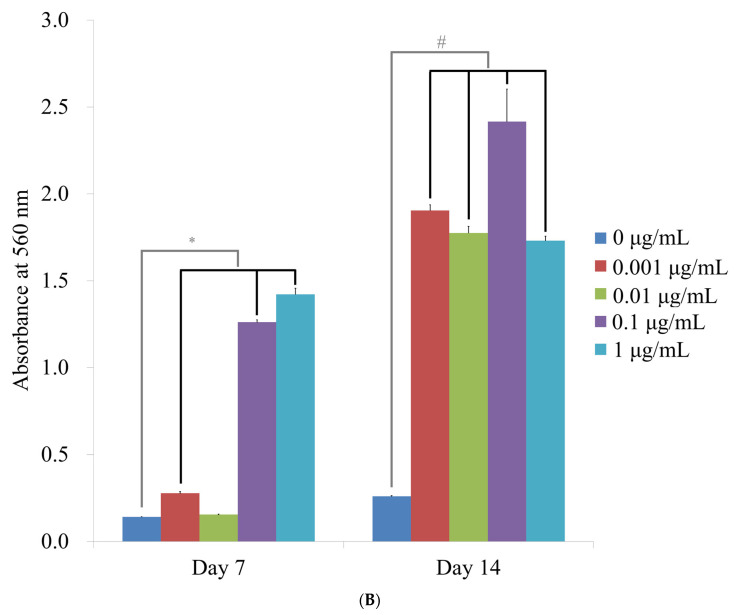
(**A**) Microscopic evaluation of Alizarin Red S staining on days 7 and 14 (original magnification ×100). Scale bars indicate 200 μm. (**B**) Quantitative analysis of Alizarin Red S staining. * Statistically significant increases were noted when compared with CCT at 0 μg/mL on day 7 (*p* < 0.05). # Statistically significant differences were seen when compared with CCT at 0 μg/mL on day 14 (*p* < 0.05).

The results of the Alizarin Red S staining on the 7th and 14th day are shown in Figure 5A. The absorbance values at 560 nm in cells cultured with 0, 0.001, 0.01, 0.1 and 1 μg/mL CCT on day 7 were 0.142 ± 0.001, 0.278 ± 0.010, 0.155 ± 0.003, 1.261 ± 0.014 and 1.422 ± 0.034, respectively (*p* < 0.05) (Figure 5B). The absorbance values of the tested cells cultured with 0, 0.001, 0.01, 0.1 and 1 μg/mL CCT on day 14 were 0.261 ± 0.003, 1.904 ± 0.032, 1.775 ± 0.037, 2.416 ± 0.187 and 1.731 ± 0.025, respectively. There was a significant increase in mineralization with the addition of CCT (*p* < 0.05).

### 3.5. Evaluation of RUNX2, BSP, OCN and COL2A1 mRNA by Quantitative Real-Time PCR

**Figure 6:**
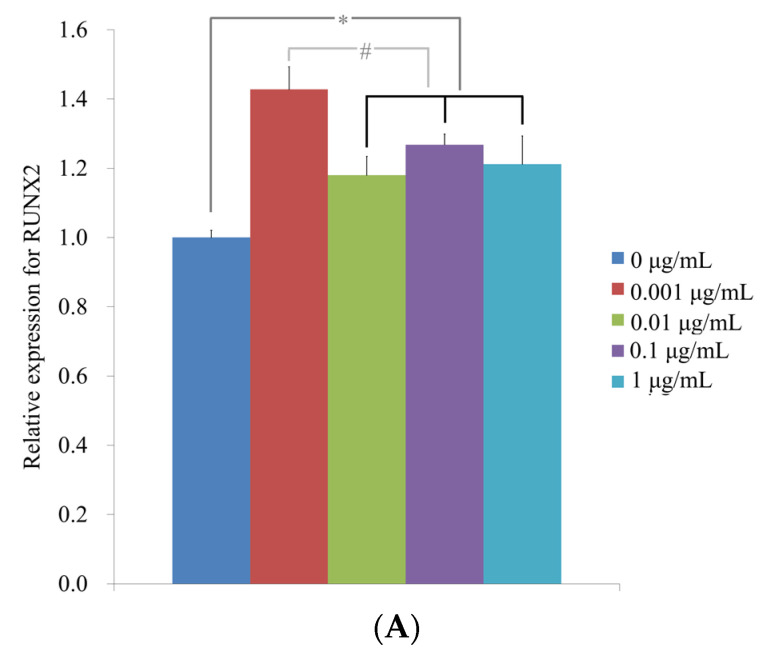

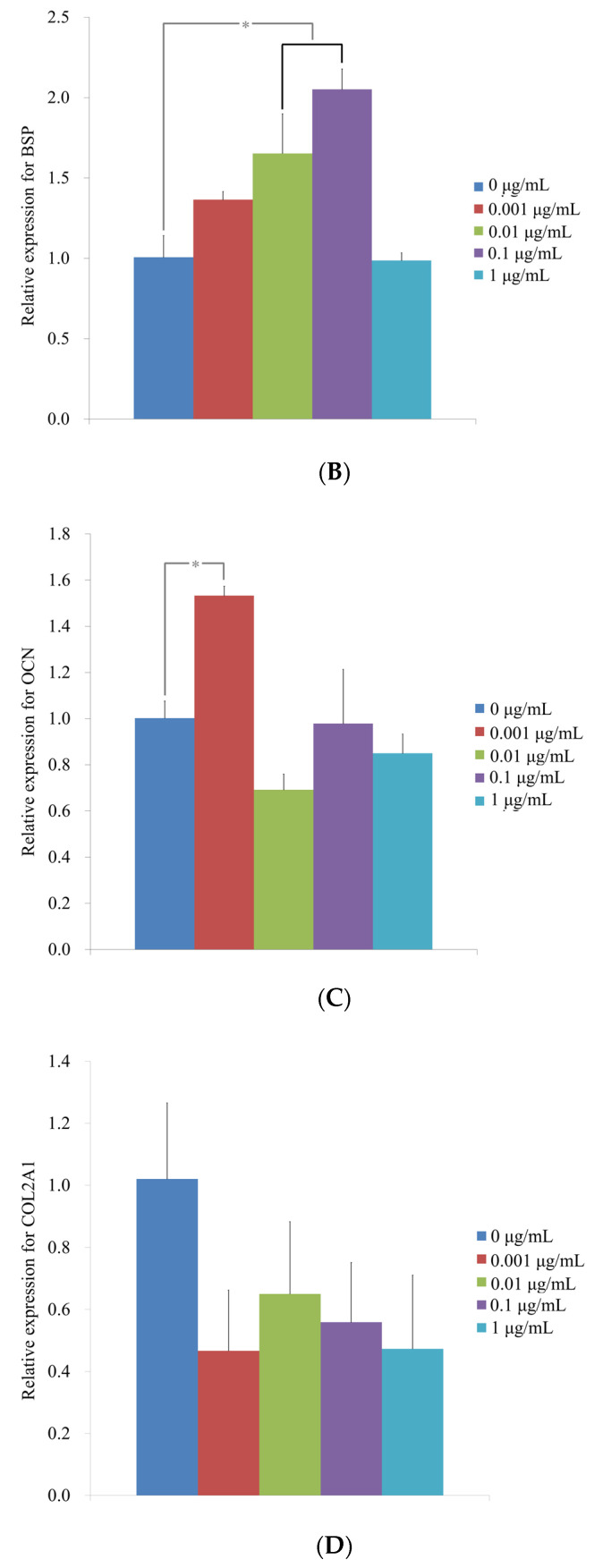
(**A**) Quantification of expression of RUNX2 mRNA by real-time polymerase chain reaction on day 3. * Statistically significant differences compared with CCT at 0 μg/mL (*p* < 0.05). # Statistically significant differences compared with CCT at 0.001 μg/mL (*p* < 0.05). (**B**) Quantification of expression of BSP mRNA by real-time polymerase chain reaction on day 7. * Statistically significant differences compared with CCT at 0 μg/mL (*p* < 0.05). (**C**) Quantification of expression of OCN mRNA by real-time polymerase chain reaction on day 7. * Statistically significant differences compared with CCT at 0 μg/mL (*p* < 0.05). (**D**) Quantification of expression of COL2A1 mRNA by real-time polymerase chain reaction on day 7.

Quantitative real-time PCR revealed that the mRNA levels of RUNX2 on day 3 were 1.00 ± 0.02, 1.43 ± 0.07, 1.18 ± 0.05, 1.27 ± 0.03 and 1.21 ± 0.08 in cells treated with CCT 0, 0.001, 0.01, 0.1 and 1 μg/mL, respectively (Figure 6A). There was a significant increase in RUNX2 expression with the addition of CCT (*p* < 0.05). Quantitative real-time PCR revealed that the mRNA levels of BSP on day 7 were 1.01 ± 0.13, 1.36 ± 0.05, 1.65 ± 0.25, 2.05 ± 0.13 and 0.99 ± 0.05 when cells were treated with CCT 0, 0.001, 0.01, 0.1 and 1 μg/mL, respectively (Figure 6B) (*p* < 0.05). Quantitative real-time PCR revealed that the mRNA levels of OCN on day 7 were 1.00 ± 0.07, 1.53 ± 0.04, 0.69 ± 0.07, 0.98 ± 0.23 and 0.85 ± 0.08 when treated with CCT 0, 0.001, 0.01, 0.1 and 1 μg/mL, respectively (Figure 6C) (*p* < 0.05). Quantitative real-time PCR revealed that mRNA levels of COL2A1 on day 7 were 1.02 ± 0.25, 0.47 ± 0.20, 0.65 ± 0.23, 0.56 ± 0.19 and 0.47 ± 0.24 in cells treated with CCT 0, 0.001, 0.01, 0.1 and 1 μg/mL, respectively (Figure 6D) (*p* > 0.05).

## 4. Discussion

This study examined the effects of different concentrations of CCT on the osteogenic differentiation of stem cells derived from human bone marrow. The results clearly showed that the application of CCT influenced the osteogenic differentiation of stem cells by regulating the expressions of RUNX2, BSP and OCN.

In general, no significant decrease was noted for cellular viability, indicating that the CCT in the tested concentration did not seem to produce adverse effects [24]. Bone regeneration is an area of great interest [25]. Osteogenic differentiation consists of several stages including proliferation, matrix maturation and mineralization [26]. Alkaline phosphatase activity is known to be the initial marker of osteogenic differentiation [27]. In this study, the application of CCT showed a significant impact on the mineralization of stem cells. Our results indicated that CCT affects the later stage of osteogenic differentiation [11]. Expression levels of various genes were tested to evaluate osteogenic capability including those of RUNX2, BSP, OCN and COL2A1 [23,28]. RUNX2 is known to be one of the key transcription factors related with osteogenic differentiation [29]. The secretion of BSP is considered to be the marker of the start of the osteogenic differentiation and BSP knockout led to little or no expression of osteogenic markers without mineralized colonization [30,31]. OCN is also considered as a bone-specific marker and it is reported to be associated with the maturation of osteogenesis [28]. COL2A1 is reported to be one of the cartilage-specific genes and no significant changes were noted in this experiment [32].

We used a wide range of concentrations to determine the appropriate capacity. The dosage may affect the effects of cumin on various situations [33]. In a previous study, 100 to 10,000 μg/mL of aqueous extract of ultra-filtrated cumin seed was applied for the evaluation of degranulation and cell viability of rat basophilic leukemia cells [34]. The obtained minimum inhibitory concentration for cumin was 2–4 µL/mL (mean: 3.1 µL/mL) [8]. The rats were gavaged with essential oil from cumin at dose levels of 0, 250, 500 and 1000 mg/kg/day and an increase in serum levels of alanine transaminase was observed only at a dose level of 1000 mg/kg/day [35]. In this study, 0.1 and 1 µg/mL of CCT was able to increase the value of the mineralization assay.

Various extraction methods have been used for cumin including extraction methods using water, methanol, ethanol and ethyl acetate [34,36,37,38,39]. An aqueous extract of cumin was applied for the evaluation of the inflammatory response in immune cells and antiallergic effects [34,40]. A methanol extract of cumin was used for the evaluation of the protective role of cumin against biomolecular damage and neuropharmacological activities [36,39]. An ethanol extract of cumin was previously shown to lower triglycerides in diabetic rats [41]. An ethyl acetate extract of cumin was tested for wound healing application [20].

The underlying mechanisms have to be evaluated further. Cumin is reported to contain various components including cuminaldehyde, terpenoids, cemene, paracymene, linalool and glucopyranoside [2,42]. Cuminaldehyde from cumin extracts exerts antinociceptive and antineuropathic effects through the involvement of opioid receptors, the L-arginine/NO/cGMP pathway and the anti-inflammatory function [43]. The anti-inflammatory effect of cumin was thought to involve nuclear factor κB and mitogen-activated protein kinase [44]. Moreover, activations of RAW264.7 and NK-92 cells occurred through nuclear factor-κB and MAPK signal pathways as indicated by the presence of phosphorylated nuclear factor-κB, ERK, JNK and p38 proteins [40].

## 5. Conclusions

In conclusion, our results showed that the application of CCT enhanced the osteogenic differentiation of mineralization in stem cells derived from bone marrow by regulating the expressions of RUNX2, BSP and OCN without affecting cellular viability. Thus, the use of CCT may produce beneficial effects on the mineralization of stem cells.

## Data Availability

All data are contained within the article.
